# Identification of tanshinone I as cap-dependent endonuclease inhibitor with broad-spectrum antiviral effect

**DOI:** 10.1128/jvi.00796-23

**Published:** 2023-09-21

**Authors:** Xiaoxue He, Fan Yang, Yan Wu, Jia Lu, Xiao Gao, Xuerui Zhu, Jie Yang, Shuwen Liu, Gengfu Xiao, Xiaoyan Pan

**Affiliations:** 1 State Key Laboratory of Virology, Wuhan Institute of Virology, Center for Biosafety Mega-Science, Chinese Academy of Sciences, Wuhan, China; 2 The Second Clinical Medical College, Jinan University (Shenzhen People’s Hospital), Shenzhen, China; 3 Savaid Medical School, University of the Chinese Academy of Sciences, Beijing, China; 4 NMPA Key Laboratory for Research and Evaluation of Drug Metabolism, Guangdong Provincial Key Laboratory of New Drug Screening, School of Pharmaceutical Sciences, Southern Medical University, Guangzhou, China; 5 Guangdong Provincial Key Laboratory of New Drug Screening, Guangzhou Key Laboratory of Drug Research for Emerging Virus Prevention and Treatment, School of Pharmaceutical Sciences, Southern Medical University, Guangzhou, China; University of Kentucky College of Medicine, Lexington, Kentucky, USA

**Keywords:** cap-snatching, endonuclease, tanshinone, severe fever with thrombocytopenia syndrome virus, broad-spectrum antivirals

## Abstract

The cap-snatching mechanism mediated by cap-dependent endonuclease, which is common among the negative-stranded, segmented RNA viruses in *Orthomyxoviridae*, *Bunyaviridae,* and *Arenaviridae*, is crucial for viral transcription and replication and is thus an attractive target for antiviral drug development. Herein, tanshinone I and its analog tanshinone IIA were identified as candidate compounds with broad-spectrum antiviral activities against bandaviruses, including severe fever with thrombocytopenia syndrome virus, Heartland virus, and Guertu virus. Additionally, the broad-spectrum antiviral activity was observed in influenza A virus and arenavirus. Further study demonstrated that tanshinone I exhibited potent antiviral activity *in vitro* and significantly reduced the viral loads *in vivo*. The underlying mechanism was speculated to involve tanshinone I binding to the active pocket of the L protein endonuclease domain to inhibit cap cleavage. This study reports candidate broad-spectrum antiviral compounds against negative-stranded, segmented RNA viruses, highlighting the endonuclease involved in the cap-snatching process as a reliable antiviral target for discovering broad-spectrum antivirals.

**IMPORTANCE:**

The spread of avian-borne, tick-borne, and rodent-borne pathogens has the potential to pose a serious threat to human health, and candidate vaccines as well as therapeutics for these pathogens are urgently needed. Tanshinones, especially tanshinone I, were identified as a cap-dependent endonuclease inhibitor with broad-spectrum antiviral effects on negative-stranded, segmented RNA viruses including bandavirus, orthomyxovirus, and arenavirus from natural products, implying an important resource of candidate antivirals from the traditional Chinese medicines. This study supplies novel candidate antivirals for the negative-stranded, segmented RNA virus and highlights the endonuclease involved in the cap-snatching process as a reliable broad-spectrum antiviral target.

## INTRODUCTION

Severe fever with thrombocytopenia syndrome (SFTS) with its typical clinical manifestations, including high fever, leukopenia, thrombocytopenia, and hemorrhage, accompanied by gastrointestinal, respiratory, and neurological symptoms, was first reported in 2009 (1, 2). Its pathogen was subsequently identified as severe fever with thrombocytopenia syndrome virus (SFTSV), a new member of *Bandavirus*, *Phenuiviridae*, and *Bunyavirales*, in 2011 (1). SFTSV continually appears in Japan (3), Korea (4), and Southeast Asia (5), and confirmed cases have also been reported in Pakistan (6) and the United States (7). SFTS is a tick-borne zoonosis that transmits to humans mainly through tick bites, and close contact interpersonal transmission and transmission through animals such as cats and dogs have also been reported (8, 9). From 2010 to 2019, a total of 13,824 cases of SFTS were reported in China, including 713 deaths, with an average mortality of 5.2% (10). To date, there are no specific prevention or treatments licensed; therefore, the World Health Organization lists SFTS as a disease in urgent need of research and development (11). In addition to SFTSV, Heartland virus (HRTV), which was first found in Missouri, United States, and Guertu virus (GTV), which is found in Xin Jiang Province, China, also reside in ticks and pose a public health threat to the world (12).

SFTSV is an enveloped, segmented ssRNA (−) virus with spherical particles of approximately 80–120 nm. Its genome consists of small (S), medium (M), and large (L) segments; the S segment encodes nucleoprotein (NP) and nonstructural proteins (NSs); the M segment encodes the envelope glycoproteins, glycoprotein N (Gn), and glycoprotein C (Gc); and the L segment encodes the L protein, which mediates viral RNA replication and transcription (13). The L protein contains an endonuclease (EndoN) domain in the N-terminal region, an RNA-dependent RNA polymerase (RdRp) domain in the middle region, and a cap-binding domain (CBD) in the C-terminal region (14). Notably, SFTSV transcription relies on an unusual “cap-snatching” mechanism shared by negative-stranded, segmented RNA viruses from *Orthomyxoviridae*, *Bunyaviridae*, and *Arenaviridae* (15). Specifically, cap-snatching is a transcription initiation process during which a nucleotide sequence between 10 and 20 nt in size is cleaved from the 5′ end of host mRNAs by the EndoN in conjunction with the CBD, and the cleaved oligonucleotide containing the 5′ cap structure is used as a primer for viral RNA transcription, leading to the synthesis of capped, translatable viral mRNAs (16).

Therefore, EndoN, which has a core fold and divalent cation-binding residues that are characteristic of PD…D/E…K nuclease superfamily (17, 18), is an attractive viral target for broad-spectrum antiviral drug development (19). Notably, baloxavir (BXA) is an effective EndoN inhibitor with superior anti-influenza efficacy in both preclinical and clinical studies (20, 21) and was also reported to exert an anti-SFTSV effect *in vitro* by inhibiting the enzymatic activity of SFTSV EndoN (22). Other reported inhibitors acting on the post-infection stage of SFTSV are favipiravir (T-705), ribavirin, calcium channel blockers, and so on (23). However, their clinical effectiveness on SFTS is difficult to determine (23). Considering the nonnegligible potential for inducing drug resistance and the risk of teratogenicity and embryotoxicity of nucleoside analogs targeting RdRp (24), new drugs targeting viral proteins of SFTSV need to be intensively investigated.

In this study, a mini library of natural products with pharmacological activity was screened to identify inhibitors against SFTSV. One of them, tanshinone I, a component of traditional Chinese medicine (TCM) for cardiovascular disease (25 – 27), was found to inhibit SFTSV replication at the post-infection stage. Further study demonstrated that its analog, tanshinone IIA, has a similar anti-SFTSV effect. Moreover, the *in vivo* efficacy of tanshinone I against SFTSV, influenza A virus (IAV), and arenavirus was confirmed in rodents. Finally, tanshinone I and IIA were proven to inhibit the enzymatic activity of EndoN, as BXA did, thus exerting a broad-spectrum antiviral effect on viruses in *Bunyaviridae*, *Orthomyxoviridae,* and *Arenaviridae*. This study reports natural products as candidate broad-spectrum antivirals and further confirms EndoN as a reliable viral target for drug development.

## RESULTS

### Hit compounds were identified from a natural product library against SFTSV

**Fig 1:**
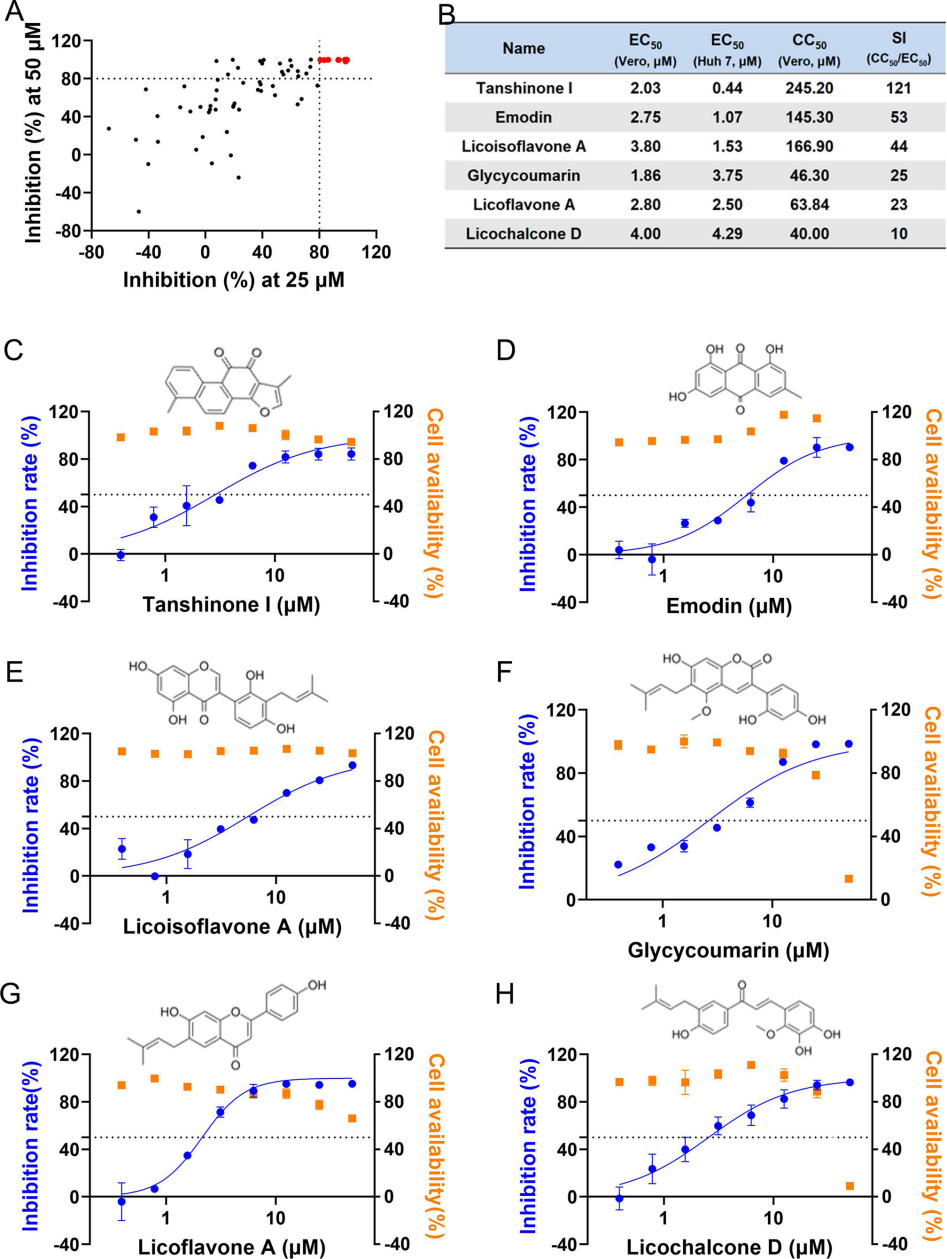
Screening hit compounds from a mini natural product library against SFTSV. (A) Identification of candidates with inhibition rates >80% at 25 µM and inhibition rates >90% at 50 µM against SFTSV on Vero cell model detected by qRT-PCR. (B) EC_50_ detected by qRT-PCR and CC_50_ detected by CCK8 assay in Vero or Huh 7 cell lines at 36 hours after treatment. SIs of the six hit compounds selected from the validation screening were listed. (C–H) Chemical structures and dose-response curves of both the antiviral activity and cytotoxicity of the hit compounds tanshinone I, emodin, licoisoflavone A, glycycoumarin, licoflavone A, and licochalcone D on Vero cells.

To find novel candidate compounds against SFTSV, qRT-PCR-based viral copy detection was employed to screen a small natural product library as described in Fig. S1A. As shown in Fig. S1B, six compounds were finally selected from the 71 compounds based on narrow thresholds for both antiviral activity and cytotoxicity. The inhibition rates at both 25 and 50 µM are collectively summarized in Fig. 1A.

Six hit compounds, tanshinone I, emodin, licoisoflavone A, glycycoumarin, licoflavone A, and licochalcone D, were subjected to antiviral assays to confirm their definite activities. Their antiviral activities were successively tested on both Vero and Huh 7 cell models by qRT-PCR, and their cytotoxicity to Vero cells was detected by CCK8 assays. As shown in Fig. 1B, the half effective concentration (EC_50_) values of the six hit compounds for both Vero and Huh 7 cells ranged from micromolar to submicromolar levels, while their half cytotoxic concentration (CC_50_) values for Vero cells varied from 40 to 245 µM. Thus, the hit compounds had a wide range of selectivity indexes (SIs, CC_50_/EC_50_), varying from 10 to 121. The chemical structures of the six hit compounds are shown at the top of the dose-response curves in Fig. 1C through H. Moreover, their inhibition of SFTSV infection in Vero cells was detected by indirect immunofluorescence assay (IFA), and decreased SFTSV infection was observed with increasing concentrations of the compounds (Fig. S1C). Among the six, tanshinone I exhibited strong antiviral activity while having the lowest cytotoxicity, thus exhibiting the highest SI at 121, which should be studied further.

### Tanshinone I inhibited SFTSV at the post-infection stage

**Fig 2:**
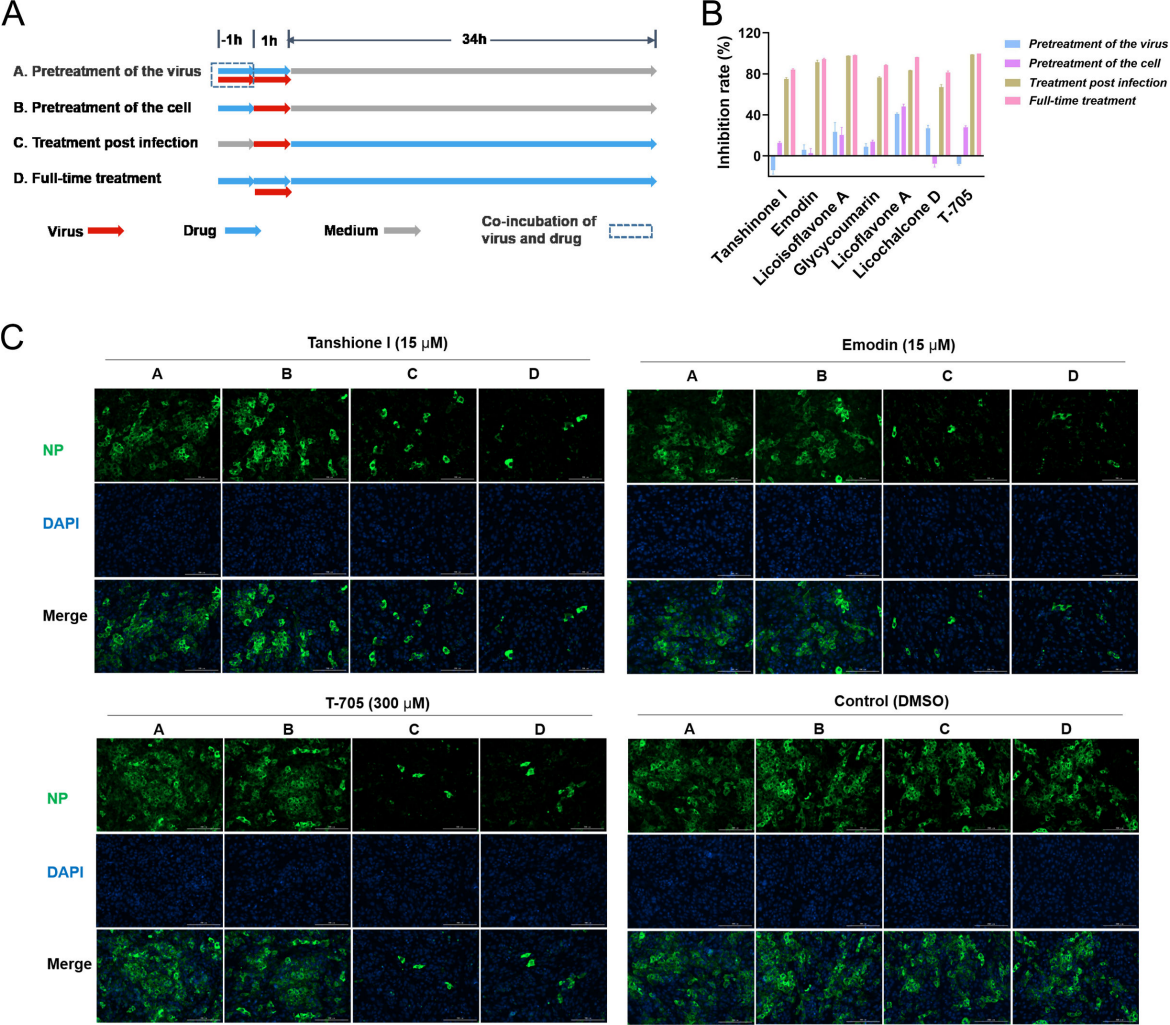
Confirmation of the hit compounds working at the post-infection stage. (A) The mode of different treatments was shown as the schematic. A, pretreatment of the virus; B, pretreatment of the cell; C, treatment post infection; and D, full-time treatment. (B) The inhibition of SFTSV after the four different treatments with the six hit compounds was detected by qRT-PCR after 36 hours. T-705 was used as a control. (C) IFA analysis of Vero cells infected with SFTSV at 36 hours after diverse treatments (A, B, C, and D, as described in panel A) with tanshinone I, emodin, T-705, or DMSO (control). Bars represent 200 µm.

A time-of-addition experiment was performed to preliminarily investigate the mechanism by which the six hit compounds inhibit SFTSV infection in terms of their ability to disrupt virion stability, block viral entry, and inhibit viral genome replication or viral budding at the post-entry stage. To achieve this goal, four patterns of treatment were carried out as described in Fig. 2A. As a GTP competitor of the viral polymerase acting at the viral genome replication stage (28), T-705 was selected as the positive control in this study since it has certain antiviral activity against SFTSV *in vitro*, and its EC_50_ was approximately 47 µM in our experimental system (Fig. S2). As shown in Fig. 2B, T-705 exhibited a comparable inhibition rate under the post-infection and full-time treatment patterns but had a negligible influence on the virions or at the entry stage. Among the six hit compounds, five, including tanshinone I, acted at the post-entry stage, as T-705 did, except licoflavone A, which acted at both the entry and post-entry stages. The inhibitory effects of tanshinone I and emodin as well as T-705 were also analyzed by IFA. As shown in Fig. 2C, the negative control (dimethylsulfoxide [DMSO]) did not influence viral infection under any treatment patterns, while tanshinone I and emodin had significant inhibitory activities under the post-entry and full-time treatment patterns, similar to T-705. These results demonstrated that tanshinone I, one of the hit compounds, exerted antiviral activity against SFTSV at the post-entry stage.

### Tanshinone I and IIA targeted the L endonuclease domain to inhibit bandaviruses, including SFTSV

**Fig 3:**
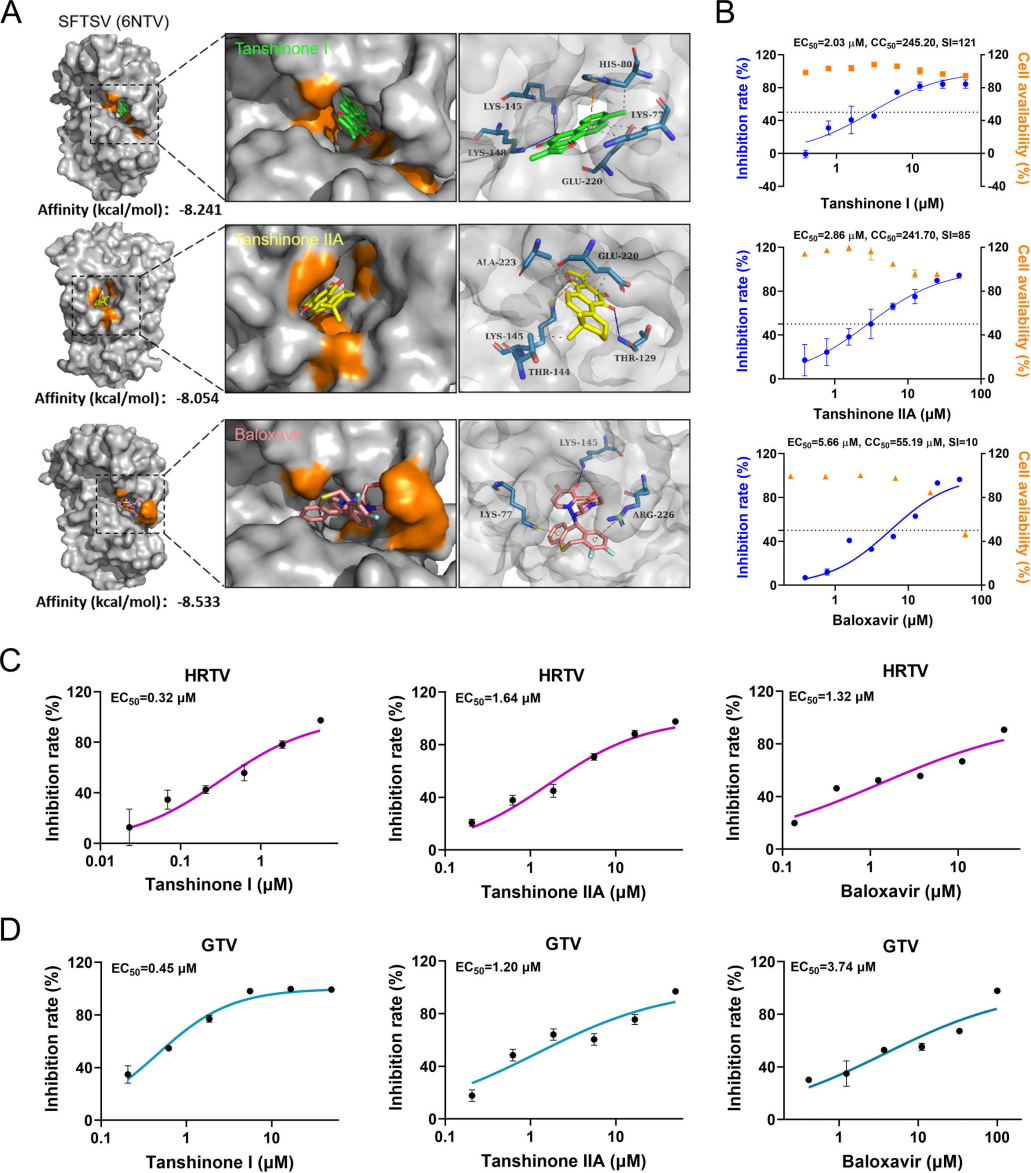
The broad-spectrum antiviral effect of tanshinone I and IIA on SFTSV, HRTV, and GTV. (A) Docking analysis of tanshinone I (green) and tanshinone IIA (yellow) on the L endonuclease domain of SFTSV (PDB:6NTV). Baloxavir (pink) was used as a control. The calculated binding energy of tanshinone I, tanshinone IIA, and baloxavir to SFTSV EndoN is shown under each structure. The binding pockets and sites are shown as either orange areas or blue sticks. (B) Both the antiviral activity and cytotoxicity of tanshinone I and tanshinone IIA on Vero cells. Calculated SIs are shown along with EC_50_ and CC_50_. Baloxavir was used as a control. (C and D) The antiviral effect of tanshinone I, tanshinone IIA, and baloxavir on HRTV and GTV in Vero cells was detected by qRT-PCR after treatment for 36 hours. EC_50_ values are shown in the figure.

The cap-snatching mechanism depends on the L EndoN domain, which initiates the transcription and genome replication of negative-stranded, segmented RNA viruses and is crucial for the replication of bandaviruses such as SFTSV, HRTV, and GTV. Therefore, molecular docking analysis was first employed to predict the binding of tanshinone I to SFTSV EndoN. BXA, previously reported to exert an anti-SFTSV effect by targeting SFTSV EndoN (22), was used as the positive control. As shown in Fig. 3A, tanshinone I (−8.241 kcal/mol) has an affinity for SFTSV EndoN (6NTV) similar to that of BXA (−8.533 kcal/mol) when docked into the active pocket of 6NTV in the same configuration. As expected, the analog of tanshinone I, tanshinone IIA, also has a high affinity (−8.054 kcal/mol) similar to that of tanshinone I and BXA. Notably, in docking analysis, tanshinone I and IIA as well as BXA bound the same residue, K145, which is one of the key sites in the PD…D/E…K nuclease superfamily motif. These suggest the potential binding of tanshinone I to SFTSV EndoN.

To test our hypothesis, a set of antiviral assays were conducted. Tanshinone IIA was found to have an EC_50_ of 2.86 µM against SFTSV and an SI of 85 in Vero cells, while the EC_50_ values of tanshinone I and BXA were 2.03 and 5.66 µM, respectively (Fig. 3B). Because HRTV and GTV belong to the *Bandavirus* genus along with SFTSV, their EndoNs contain the highly conserved PD-(D/E)-X-K nuclease superfamily motif (H…PD…D/E…K) (29); hence, they have high structural and functional homology. Thus, the broad-spectrum antiviral effects of tanshinone I and IIA on bandaviruses were explored on these viruses. As a result, both tanshinone I and IIA effectively inhibited HRTV and GTV with EC_50_s at the micromolar level, as shown in Fig. 3C and D. BXA had EC_50_ values of 1.32 and 3.74 µM against HRTV and GTV, respectively. These results collectively indicated the broad-spectrum antiviral activity of tanshinone I and IIA as well as BXA on bandaviruses, suggesting the underlying principle of targeting EndoN to exert antiviral effects.

### Tanshinone I and IIA target EndoN to inhibit enzymatic activity

**Fig 4:**
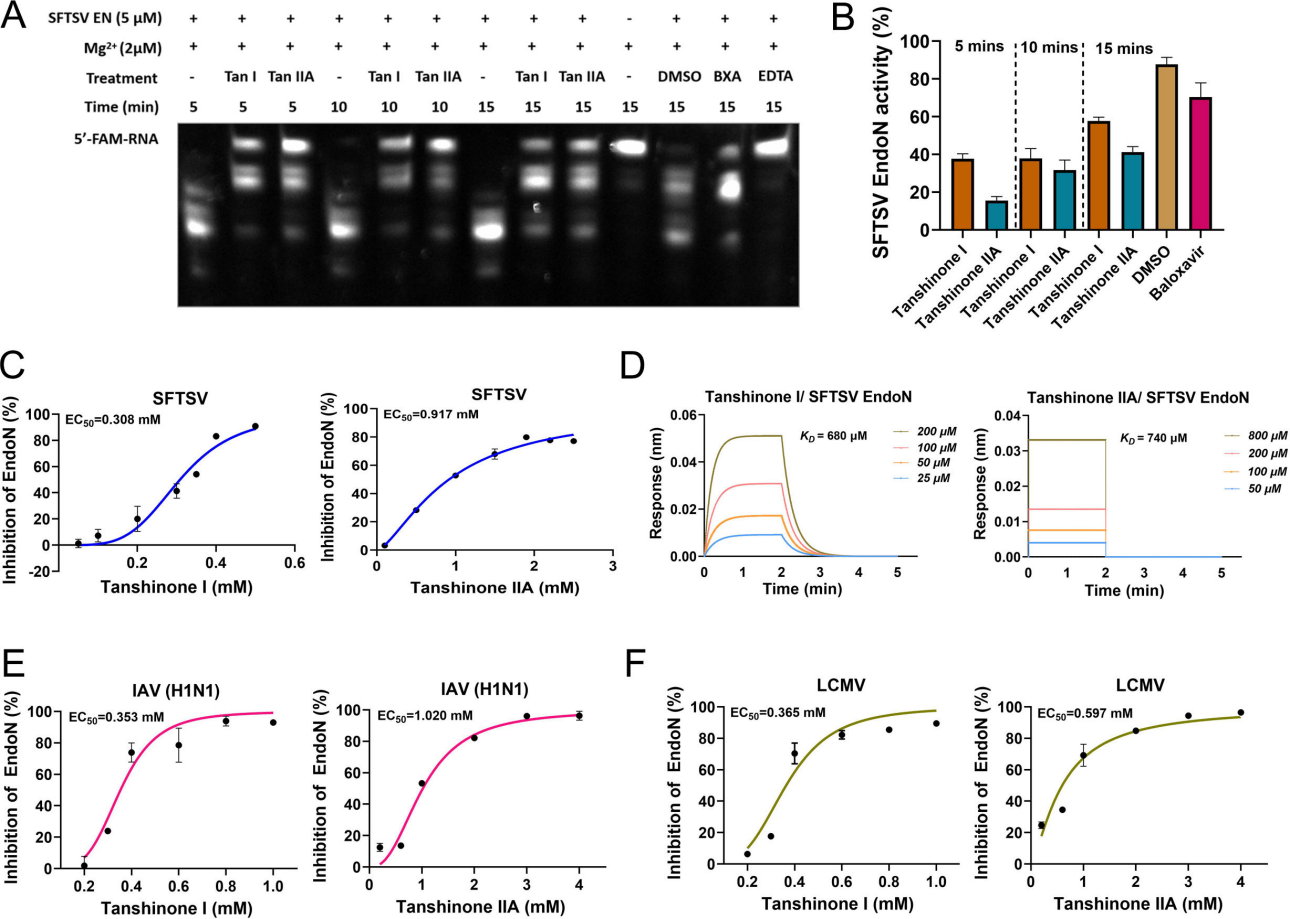
The action of tanshinone I and tanshinone IIA on EndoNs. (A) The inhibition of the enzymatic activity of SFTSV EndoN by tanshinone I (0.5 mM) and tanshinone IIA (2 mM) at 5, 10, and 15 minutes was detected by gel electrophoresis. Baloxavir (10 mM), DMSO (1%), and EDTA (50 µM) were used as the controls. (B) Intensity analysis of the top bands from the image in panel A. (C, E, and F) Dose-dependent inhibition of the enzymatic activity of SFTSV, IAV, and LCMV EndoNs by tanshinone I or IIA was detected by FRET assays. (D) The affinity of tanshinone I and IIA to SFTSV EndoN was detected by BLI, *K_D_* s were calculated and shown with the fitted curves.

To find more evidence that tanshinone I and IIA act on EndoN, enzymatic activity assays and molecular interaction analysis were carried out. First, a set of enzymatic activity assays analyzed by gel electrophoresis were conducted with tanshinone I and IIA at fixed concentrations, in which DMSO, BXA, and EDTA were used as the negative control, positive control, and system reference, respectively. As shown in Fig. 4A, both tanshinone I and IIA significantly inhibited the cleavage of the FAM-labeled ssRNA substrates (the top bands) at 5, 10, and 15 minutes; at the same time, BXA partially inhibited the cleavage. The enzymatic activity converted from the quantity of the top band in Fig. 4A more intuitively showed the definite inhibitory effects of tanshinone I and IIA on ssRNA cleavage (Fig. 4B).

Furthermore, a fluorescence resonance energy transfer (FRET)-based enzymatic activity assay was performed to observe the dose-dependent effects of tanshinone I and IIA on EndoNs. Before starting the formal assay, a test of BXA and EDTA on SFTSV EndoN was conducted to ensure the reliability and stability of the system (Fig. S3A). In Fig. S3B, obvious dose-dependent responses were observed with the change in fluorescence signals influenced by tanshinone I and IIA before the reaction was saturated in 60 minutes (Fig. 4C). Based on the inhibition rate calculated from the fluorescence units at different working concentrations, the EC_50_ values of tanshinone I and IIA for SFTSV EndoN enzymatic activity were determined to be 0.308 and 0.917 mM, respectively. The molecular interaction assay demonstrated the binding of tanshinone I and IIA to SFTSV EndoN with *K_D_* values of 680 and 740 µM, respectively (Fig. 4D), although the association and dissociation patterns between tanshinone I and IIA were different. To our surprise, tanshinone I and IIA exhibited micromolar EC_50_ values on EndoNs from IAV (H1N1) and arenavirus (lymphatic choriomeningitis virus, LCMV), respectively (Fig. 4E and F; Fig. S3C), suggesting their extensive actions on EndoNs from different families. These results collectively demonstrated that tanshinone I and IIA target EndoN to exert antiviral effects by inhibiting the cap cleavage.

### Tanshinone I and IIA have broad-spectrum antiviral effects on cap-snatching mechanism-dependent viruses

**Fig 5:**
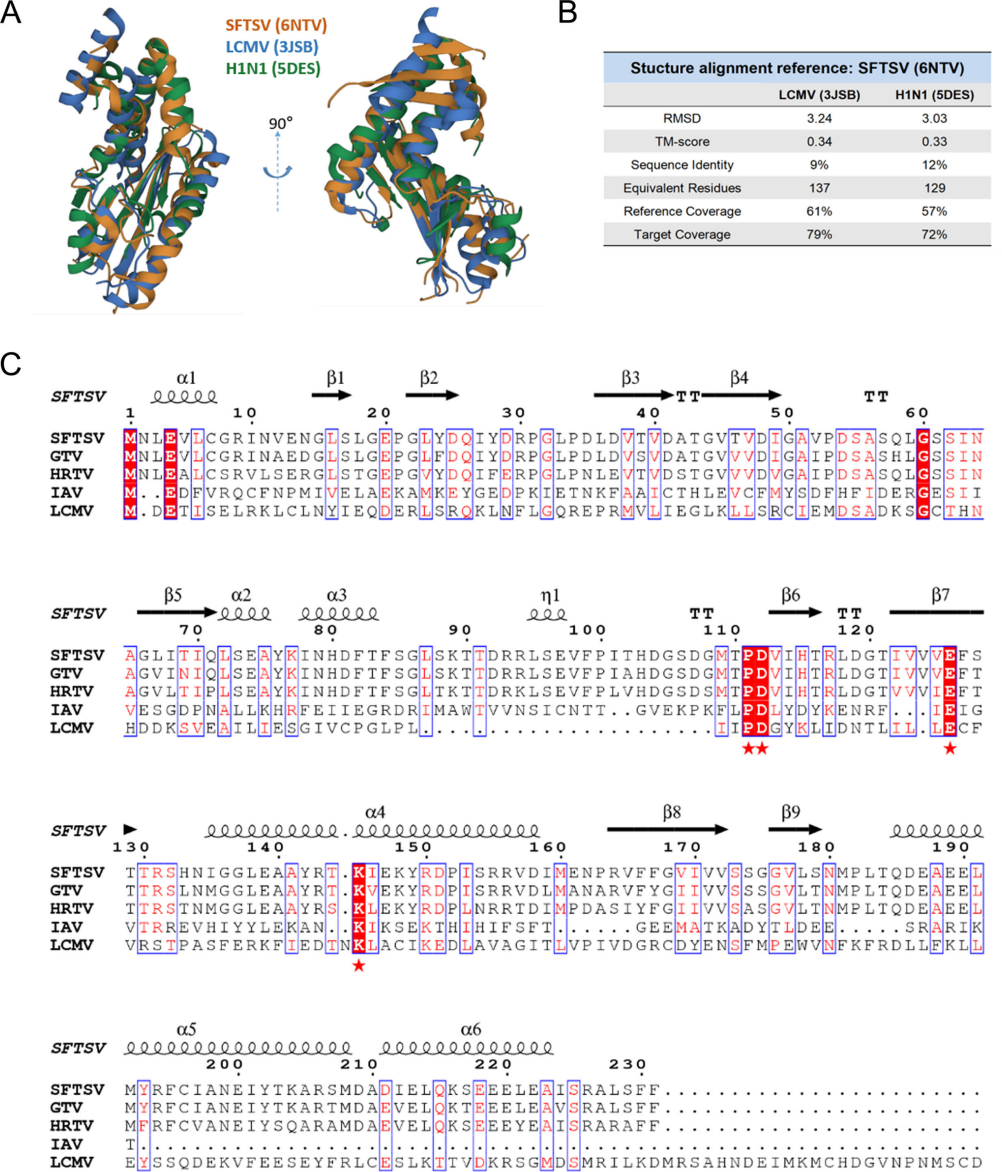
Alignment on EndoNs from *Phenuiviridae*, *Orthomyxoviridae,* and *Arenaviridae* viruses. (A) Comparison on the structures of SFTSV EndoN (PDB:6NTV, orange), LCMV EndoN (PDB:3JSB, blue), and IAV (H1N1) EndoN (PDB:5DES, green). (B) The comparison results of LCMV and IAV EndoNs are summarized in the table; SFTSV EndoN was made as a reference. (C) Analysis of the EndoN superfamily motif from SFTSV (YP_006504091.1), HRTV (YP_009047242.1), GTV (YP_009666941.1), LCMV (AFH08746.1), and IAV (H1N1) (AEA02268.1) based on their sequences. Amino acids marked with star (PD…E…K) were superfamily motif.

**Fig 6:**
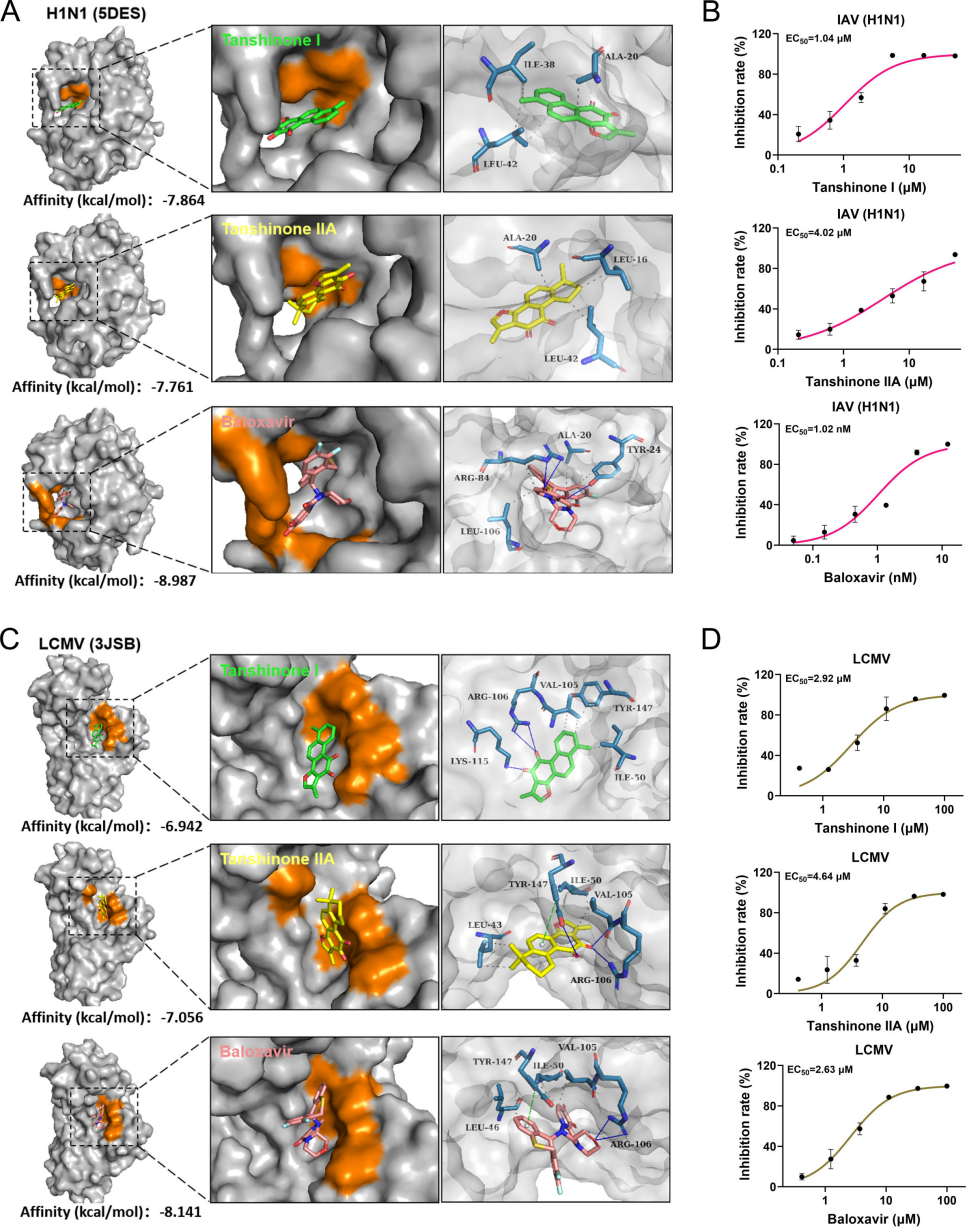
Broad-spectrum antiviral activity of tanshinone I and tanshinone IIA against IAV and LCMV. (A and C) Docking analysis of tanshinone I (green) and tanshinone IIA (yellow) to the PA N-terminal domain of IAV (H1N1) (PDB:5DES) and the L endonuclease domain of LCMV (PDB:3JSB). Baloxavir (pink) was used as a control. The calculated binding energy of tanshinone I, tanshinone IIA, and baloxavir to H1N1 and LCMV EndoNs is shown under each structure. The binding pockets and sites are shown as either orange areas or blue sticks. (B and D) The antiviral effect of tanshinone I and IIA against H1N1 in MDCK cells and LCMV in BHK-21 cells was detected by qRT-PCR, respectively; baloxavir was used as a control. EC_50_ values are shown with the curves.

Considering that the cap-snatching mechanism-dependent viruses belong to the *Orthomyxoviridae* and *Arenaviridae* in addition to *Bunyaviridae* (15), we explored the potential broad-spectrum antiviral effect of tanshinone I and IIA on representative viruses in *Orthomyxoviridae* and *Arenaviridae*, namely, IAV (H1N1 strain) and LCMV, based on the results in Fig. 4. First, an alignment of the structures of the EndoNs from SFTSV (6NTV), LCMV (3JSB), and H1N1 (5DES) was conducted, and a conserved active pocket was found among the three, despite the low identity in their sequences (Fig. 5A and B). Furthermore, the conserved PD-E-K nuclease superfamily motif was found among the protein sequences of the EndoNs from SFTSV, HRTV, GTV, IAV, and LCMV (Fig. 5C). Based on the alignment results, we conducted docking analysis of tanshinone I and IIA on the EndoNs of IAV (5DES) and LCMV (3JSB); BXA was used as a control. The affinities of tanshinone I, tanshinone IIA, and BXA to IAV EndoN were –7.864, –7.761, and −8.987 kcal/mol, respectively, and those to LCMV EndoN were –6.942, –7.056, and −8.141 kcal/mol, respectively (Fig. 6A and C), implying probable broad-spectrum binding to the EndoNs investigated in this study.

According to the above analysis results, we verified the antiviral effects of tanshinone I and IIA on IAV and LCMV *in vitro*, and BXA was used as a control. In accordance with previous reports (20, 21), BXA showed potent antiviral activity against IAV, with an EC_50_ of 1.02 nM, and the EC_50_ values of tanshinone I and IIA against IAV were 1.04 and 4.02 µM, respectively (Fig. 6B), which agree with the affinity predicted by molecular docking shown in Fig. 6A. Moreover, tanshinone I, tanshinone IIA, and BXA had EC_50_ values of 2.92, 4.64, and 2.63 µM, respectively, against LCMV (Fig. 6D). These results further defined the broad-spectrum antiviral activity of tanshinone I and IIA and emphasized the feasibility of targeting EndoNs to discover candidate broad-spectrum antiviral drugs.

### Tanshinone I and tanshinone IIA exert antiviral efficacy against SFTSV *in vivo*

**Fig 7:**
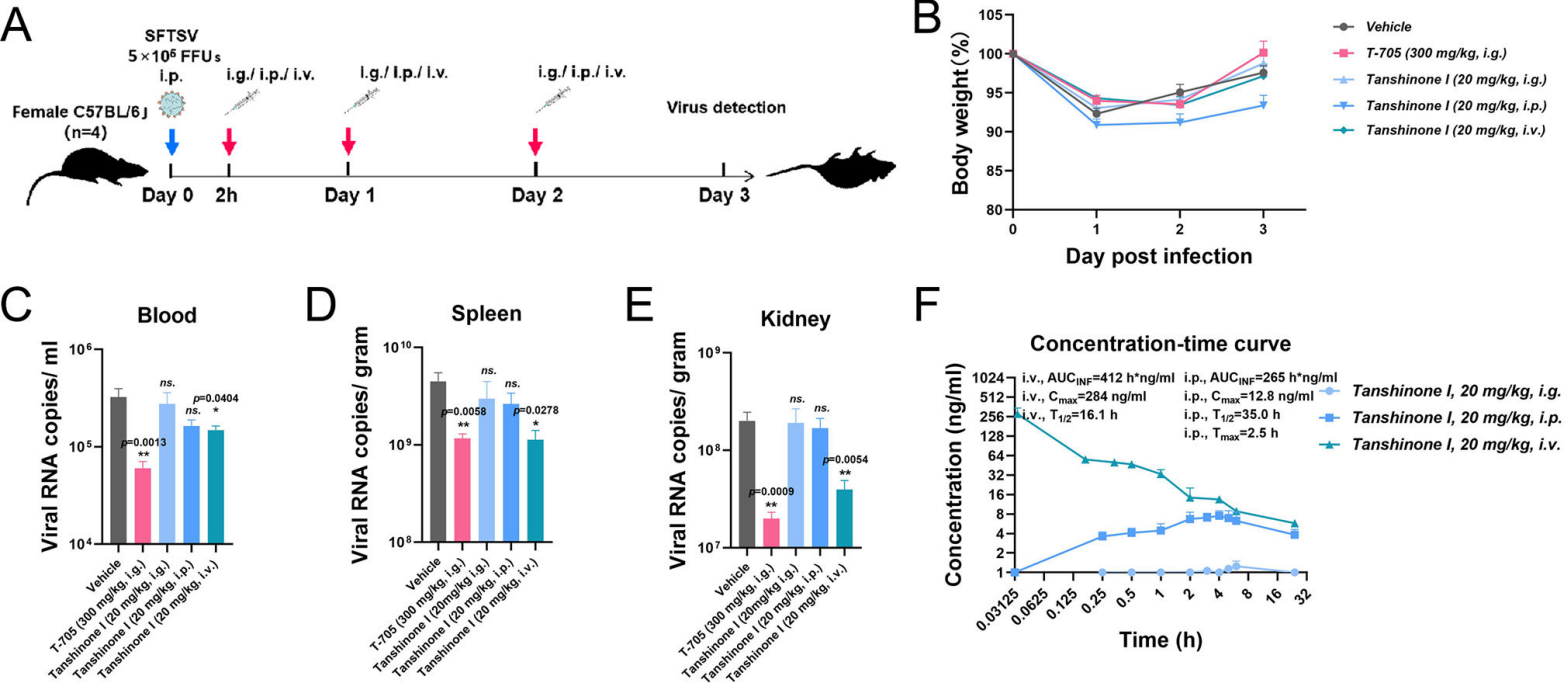
The influence of drug delivery routes on the *in vivo* efficacy of tanshinone I. (A) Scheme of the treatments for the animals. Female C57BL/6J mice (*n* = 4, 6–8 weeks) were treated with tanshinone I via i.g. administration, i.p. injection, or i.v. injection after challenge with SFTSV (5 × 10^5^ FFUs) via i.p. injection. (B) Body weight was recorded daily and graphed upon challenge. (C–E) Viral copies in blood, spleen, and kidney were measured by qRT-PCR at 3 d.p.i. (F) The pharmacokinetic data of tanshinone I administrated via i.g., i.p., and i.v. in 24 hours were collected from SD rats. The concentration-time curves were graphed from at least nine points, and the pharmacokinetic parameters were calculated and shown with the curves.

To objectively evaluate *in vivo* antiviral efficacy, we employed the adult C57BL/6J mouse model to investigate the efficacy of tanshinones against SFTSV, mainly examining the indexes of viremia and organ infection based on the report from Jin et al. (30). As described in the schematic diagram in Fig. 7A, mice were treated via intragastric (i.g.) administration, intraperitoneal (i.p.) injection, or intravenous (i.v.) injection 2 hours after the challenge with 5 × 10^5^ focus forming units (FFUs) of SFTSV via i.p. injection. Blood was collected to measure viral copy numbers 3 days post infection (d.p.i.), and the body weight was recorded daily. From the body weight curves, mild change (less than 10%) was observed no matter in vehicle group or treatment groups (Fig. 7B). When it comes to the viral copies, the administration of 20 mg/kg tanshinone I via i.g. or i.p. negligibly reduced the average viral copies in the blood, spleen, and kidney; in comparison, the administration of 20 mg/kg tanshinone I via i.v. significantly reduced the viral copies in the blood, spleen, and kidney (Fig. 7C through E). Correspondingly, the pharmacokinetic parameters reflected a poor bioavailability of tanshinone I administrated via i.g. or i.p. (Fig. 7F), which in turn emphasized that i.v. administration was necessary to maintain a relative high plasma exposure and concentration and thus ensuring an *in vivo* antiviral effect. The investigation of different delivery routes highlighted that tanshinone I has an anti-SFTSV effect *in vivo*, and its *in vivo* efficacy is affected by the route of delivery.

**Fig 8:**
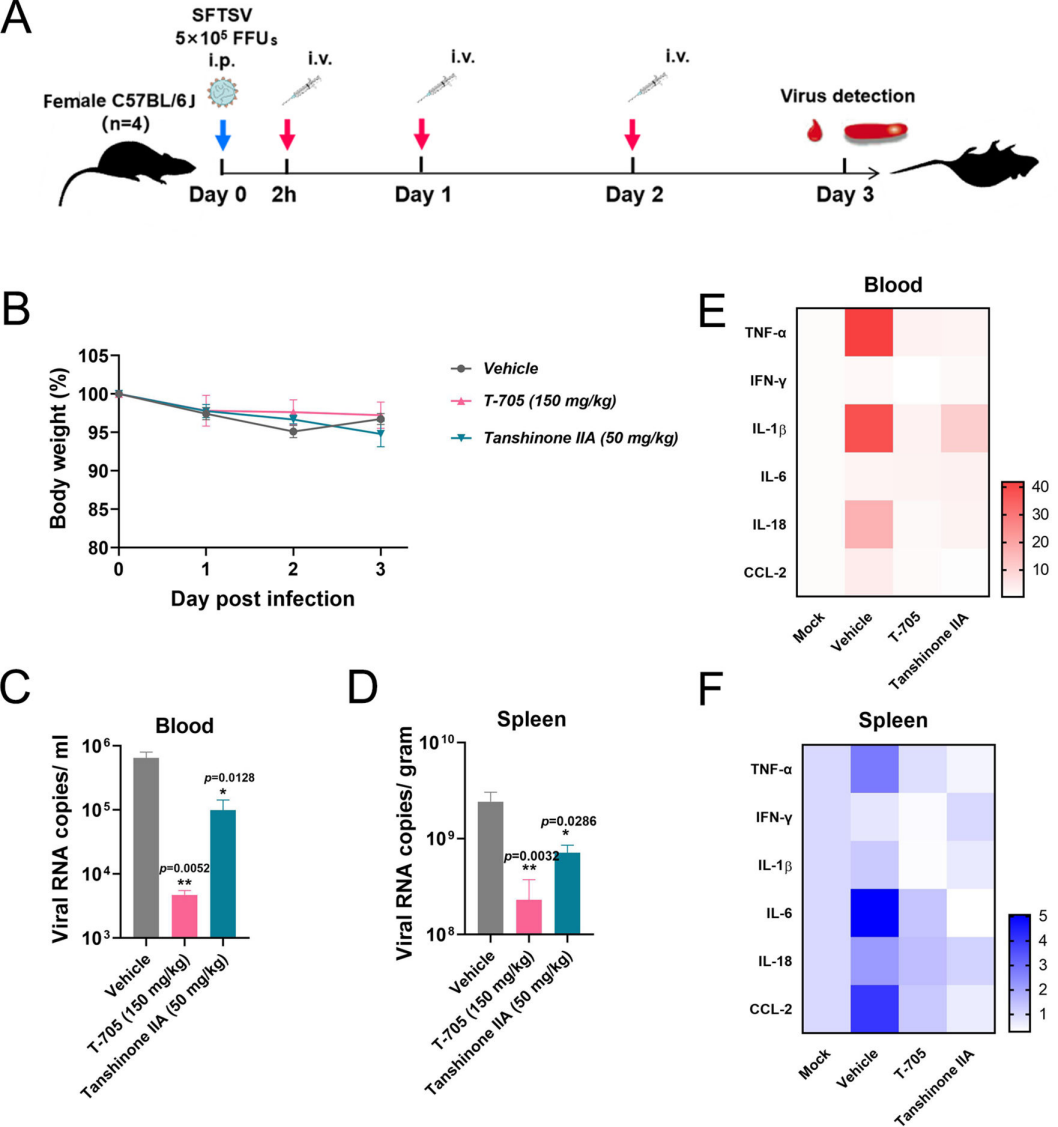
The *in vivo* antiviral efficacy of tanshinone IIA against SFTSV. (A) Scheme of the challenge and drug delivery. Female C57BL/6J mice (*n* = 4, 6–8 weeks) were treated with tanshinone IIA at 50 mg/kg via i.v. injection, and T-705 at 150 mg/kg (twice a day) via i.g. administration was used as the control. (B) The body weight curves are derived from daily recorded data. (C and D) Viral copies in both the blood and spleen were measured by qRT-PCR at 3 d.p.i. (E and F) The proinflammatory cytokines in both the blood and spleen were quantified by qRT-PCR, and the results are summarized in heatmaps and shown independently in (Fig. S4).

Subsequently, we evaluated the *in vivo* efficacy of the tanshinone I analog, tanshinone IIA, in the same model by i.v. delivery. Starting 2 hours post-challenge with 5 × 10^5^ FFUs of SFTSV via i.p. injection, mice were daily administered 50 mg/kg tanshinone IIA via i.v. or twice a day with 150 mg/kg T-705 via i.g. The blood and spleen were collected for viral copy detection at 3 d.p.i. (Fig. 8A). As shown in Fig. 8B, neither tanshinone IIA nor T-705 exhibited obvious toxicity in mice within 3 days. Compared to the vehicle, both tanshinone IIA and T-705 effectively reduced the viral copies in the blood as well as in the spleen, an organ with high blood flow (Fig. 8C and D). Moreover, the expression of representative proinflammatory factors in the blood and spleen, such as TNF-α, IL-1β, IL-6, and IL-18, which was violently stimulated by SFTSV infection, was markedly decreased by tanshinone IIA or T-705 treatment, as summarized in Fig. 8E and F and shown independently in Fig. S4A and B, further corroborating the *in vivo* anti-SFTSV efficacy of tanshinone IIA.

### Tanshinone I plays a broad-spectrum antiviral effect against IAV and LCMV *in vivo*

**Fig 9:**
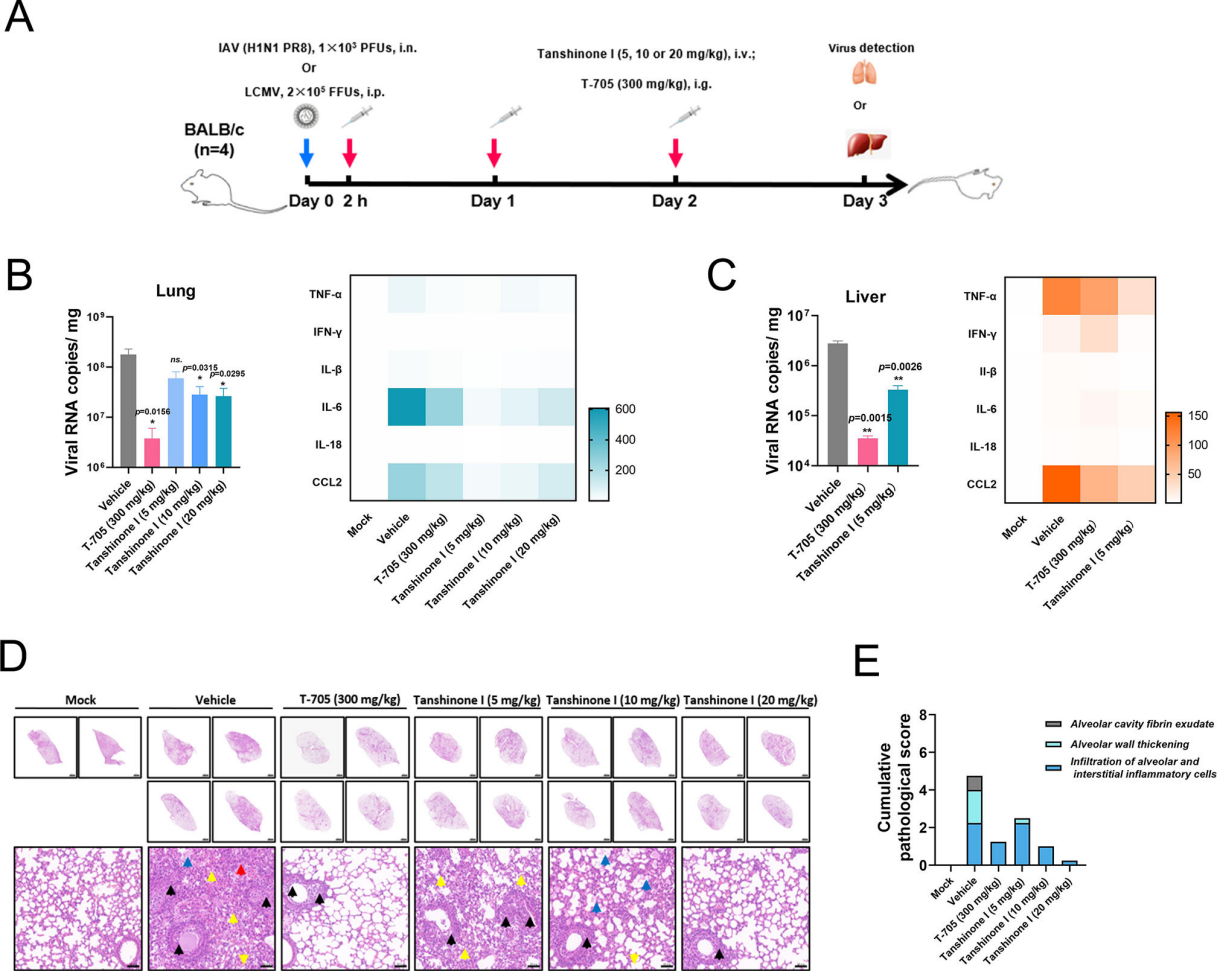
The broad-spectrum antiviral effects of tanshinone I *in vivo*. (A) Scheme of IAV and LCMV challenge and drug delivery. Male BALB/c mice (*n* = 2 or 4) were treated with tanshinone I at 5, 10, or 20 mg/kg via i.v. injection, or T-705 at 300 mg/kg via i.g. administration. Solvent was used as the vehicle. (B and C) Viral copies in the lung and liver were measured by qRT-PCR at 3 d.p.i. The corresponding proinflammatory cytokines in the lung and liver were quantified by qRT-PCR, and the results are summarized in heatmaps. (D) Pictures captured after H&E staining are displayed with full or local visual fields. The yellow arrows indicate inflammatory cells infiltrating the tissue; the blue arrows indicate a small number of fibrin-like structures; the red arrows indicate alveolar epithelial cell proliferation and alveolar wall thickening; and the black arrows indicate a small number of necrotic cells with fragmented nuclei and deep staining of solidification in the lumen of bronchioles. Bars, 1,000 and 50 µm. (E) The cumulative pathological scores from the main indexes were calculated for each group.

The *in vivo* broad-spectrum antiviral effect of tanshinone I was investigated in both IAV and LCMV challenged rodents. Following the scheme in Fig. 9A, BALB/c mice were challenged with 1 × 10^3^ PFUs of IAV (H1N1) via intranasal (i.n.) or 2 × 10^5^ FFUs of LCMV via i.p. and daily administered with 5, 10, or 20 mg/kg tanshinone I via i.v. or 300 mg/kg T-705 via i.g. Three days post challenge, the lungs or livers were collected for viral copy detection, proinflammatory factor analysis, and pathological evaluation, and body weight was recorded daily as usual. The results showed that tanshinone I had negligible influence on the body weight of 4-week BALB/c mice in the IAV challenged model or the 6-week BALB/c mice in the LCMV challenge model, similar to T-705 (Fig. S5). Moreover, tanshinone I lowered the viral copies in the lung in the IAV challenged mice at both 10 and 20 mg/kg, and downregulated the proinflammatory factors, which reflected an anti-IAV effect of tanshinone I on IAV (Fig. 9B). Similarly, tanshinone I reduced the viral copies and proinflammatory factors in the liver in the LCMV- challenged mice (Fig. 9C). From the hematoxylin and eosin-staining pictures, significant pathological remission in the lungs was observed after treatment with either T-705 or tanshinone I (Fig. 9D). The pathological score in Fig. 9E calculated from three main pathological indexes further confirmed the *in vivo* antiviral efficacy of tanshinone I.

## DISCUSSION

Transcription depending on the 5′ cap primer being cleaved from host mRNAs by viral EndoN, namely, the cap-snatching mechanism, is the initiation step of viral transcription and is widely employed by the negative-stranded, segmented RNA viruses in *Orthomyxoviridae*, *Bunyaviridae,* and *Arenaviridae* (15, 31). EndoN, as a functionally conserved enzyme among *Orthomyxoviridae*, *Bunyaviridae,* and *Arenaviridae*, is an attractive broad-spectrum target for drug development. Drugs targeting the EndoN of influenza virus, also named PA_N_, have been under development since the 1990s (18, 32). Finally, in 2018, baloxavir marboxil, whose trade name is Xofluza, was successively approved in Japan (https://www.shionogi.com/global/en/company.html) and the USA (https://www.fda.gov/news-events/press-announcements/fda-approves-new-drug-treat-influenza) as a first-in-class broad-spectrum anti-influenza drug that acts on a new viral target, EndoN, in addition to those that target neuraminidase or polymerase. In particular, BXA is a single-dose drug and is effective against oseltamivir-resistant strains (33). To our knowledge, T-705, a nucleoside analog, appeared to have higher activity than tanshinones at a high dose of 300 mg/kg, while it has the potential to induce rapid drug-resistant mutations and has a risk of teratogenicity (34); thus, it is prohibited for gravidas and infants (35), which is the recognized potential disadvantage of nucleoside analogs (36). Tanshinones were revealed as cap-dependent endonuclease inhibitors (CENi) in our study, such as baloxavir, which exhibited super antiviral activity against IAV, while its side effects on teratogenicity have rarely been reported. Thus, CENis, like tanshinones, may have an advantage in reducing side effects in terms of acting on a completely different viral target.

Herein, we screened a mini library consisting of compounds from Chinese herbs such as *Andrographis*, *Rabdosia*, *Houttuynia*, *isatidis*, honeysuckle, dandelion, *Salvia miltiorrhiza*, patchouli, *Forsythia*, *Rheum officinale*, *Rhodiola*, and licorice, which are ingredients of TCMs used to treat viral infections. We thereby found several compounds from *Salvia miltiorrhiza*, *Rheum officinale*, and licorice with good antiviral activity against SFTSV and SIs above 10, which is worth further study. Among them, tanshinone I from *Salvia miltiorrhiza* was demonstrated to have good anti-SFTSV activity at both the cellular and animal levels. Furthermore, tanshinone I and its analog tanshinone IIA were demonstrated to exert broad-spectrum antiviral effects on bunyaviruses (SFTSV, HRTV, and GTV), orthomyxoviruses (IAV), and arenaviruses (LCMV) by inhibiting the enzymatic activity of EndoN. Tanshinones, danshensu, and salvianolic acids from *Salvia miltiorrhiza* have robust pharmacological activity against cardiovascular diseases (25 – 27), while their antiviral activities have rarely been reported. In fact, we have previously reported that salvianolic acid C and danshensu target the viral entry stage to exert anti-severe acute respiratory syndrome coronavirus 2 activity (37, 38). This study reports that tanshinones exert broad-spectrum antiviral effects on bunyaviruses, orthomyxoviruses, and arenaviruses by inhibiting EndoN, thus expanding the application of *Salvia miltiorrhiza* and its ingredients and exploring the medicinal value of Chinese herbs. In addition to the natural products from *Salvia miltiorrhiza*, those from *Andrographis*, honeysuckle and dandelion are worthy of further systematic analysis.

Although a comprehensive evaluation of antiviral activity and an investigation on the potential mechanism were conducted, studies on in-depth resolution of the binding sites of tanshinone I or IIA to EndoN were difficult to follow up. One of the reasons is that tanshinones can penetrate the cellular membrane well, but their water solubility is poor, which limits the mechanism analysis at the molecular level and the *in vivo* dose optimization, thus limiting their applications. In fact, we compared i.g. administration, i.p. injection, and i.v. injection and proved that delivery via i.v. is the most effective route. Despite that, a dose higher than 20 mg/kg of tanshinone I via i.v. to investigate its activity, toxicity, or metabolism cannot be realized due solely to its poor water solubility. Sulfonated tanshinone IIA significantly improved water solubility and is in clinical trials for cardiovascular diseases (NCT01637675 and NCT02524964) in terms of its action on store-operated Ca^2+^ entry (39); however, poorer antiviral activity of tanshinone IIA sulfonate sodium against SFTSV than tanshinone I or IIA was identified (data not shown). Therefore, side effects may occur in the cardiovascular system in the absence of a definite antiviral blood concentration when applicating tanshinones. In brief, group modifications based on the chemical skeleton of tanshinone aimed at improving amphipathy as well as druggability are necessary in further studies.

EndoNs from SFTSV, IAV, and LCMV are diverse in their primary sequences but have highly conserved structures. To our knowledge, the enzymatic active pocket is constructed by acidic and basic amino acids such as PD-D/E-K as well as some important amino acids around them (40, 41). Hence, compounds that bind to the active pocket can theoretically exhibit inhibitory effects on the enzymatic activity, regardless of whether the amino acid is conserved. Certainly, compounds that bind to the amino acids that are included in the PD-D/E-K nuclease superfamily have greater potential to exert broad-spectrum effects. In our study, tanshinones could dock into the EndoN active pockets of SFTSV, IAV, and LCMV at varied sites with relatively high affinity, could bind SFTSV EndoN detected by biolayer interferometry (BLI), and displayed broad-spectrum antiviral effect both *in vitro* and *in vivo*, implying a potential broad-spectrum mechanism that has not been revealed. Resolution of definite binding sites through biochemical or co-crystallization analysis would be helpful to explain the broad-spectrum mechanism in the future study.

## MATERIALS AND METHODS

### Cells and viruses

Vero, Huh 7, BHK-21, A549, and MDCK cells were maintained in Dulbecco’s modified Eagle’s medium (DMEM; Gibco, USA) supplemented with 10% fetal bovine serum (Gibco, NY, USA) and 1% antibiotic/antimycotic (Gibco, NY, USA) at 37°C in a humidified 5% CO_2_ incubator.

SFTSV (HBMC16), HRTV (MO-4), GTV (DXM), LCMV (Armstrong), and IAV (H1N1) were obtained from the China Centre for General Virus Culture Collection. LCMV, SFTSV, HRTV, and GTV were propagated with Vero cells, LCMV was propagated with BHK-21 cells, and IAV was propagated with MDCK cells.

### Screening of natural compounds

A mini library of natural products with pharmacological activity was purchased from MCE (China). Compounds were stored in DMSO at −80°C until use. Vero cells were seeded in 48-well plates at a density of 1 × 10^5^ cells per well in a volume of 200 µL and incubated overnight. Cell monolayers were treated with the compounds at a final concentration of 50 or 25 µM for 1 hour and infected with SFTSV at an MOI of 1 for another hour. Then, the supernatants were replaced with fresh medium with the corresponding compounds. DMSO (0.1%) was added in the same way as the negative control. After incubation for an additional 36 hours, the cell supernatants from each well were collected for viral copy detection.

### Dose-response curves quantified by qRT-PCR

Monolayer cells (Vero or Huh 7 for SFTSV, HRTV, and GTV; A549 for LCMV; and MDCK for IAV) were incubated with the hit compounds for 1 hour at 37°C. Then, a 10 µL aliquot containing the assigned virus (MOI = 1 for SFTSV, GTV, and HRTV; MOI = 0.01 for LCMV and IAV) was added and incubated at 37°C for 1 hour. Next, the excess viruses were fully removed, the cells were washed with 200 µL of PBS, and 200 µL of fresh medium containing the corresponding compounds was added. Then, 150 µL of the cell supernatant was collected for detection 36 hours later. The solvent was used as the negative control (DMSO, 0.1% DMEM diluted with 2% fetal bovine serum), and T-705 was used as the positive control.

Total RNA was extracted from the cell supernatants by a DNA/RNA Extraction Kit (Vazyme, China) according to the manufacturer’s instructions. For quantitation of SFTSV vRNA, a standard curve was established with a plasmid containing the full-length NP gene, and qRT-PCR was performed according to the instructions of the HiScript II One Step qRT-PCR SYBR Green Kit (Vazyme, China). A standard curve for GTV and HRTV was based on a plasmid containing the SFTSV L protein complete sequence, a standard curve for LCMV was based on a plasmid containing the LCMV L segment sequence, and a standard curve for IAV was based on a plasmid containing the H1N1 HA sequence. The primer sequences for SFTSV, HRTV, GTV, LCMV, and IAV are listed in Table S1.

### Cytotoxicity assay

Vero cells seeded in 96-well plates overnight were treated with gradient dilutions of hit compounds for 36 hours. Next, the culture medium containing the compounds was replaced with fresh medium containing Cell Counting Kit-8 (CCK8) reagent (Vazyme, China). Then, the cells were incubated at 37°C for an additional 2 hours in the dark, and the absorbance at OD_450_ was detected by a microplate reader (SYNERGY H1, BioTek, USA). DMSO (1%) served as a control.

### Indirect immunofluorescence assay

Vero cells, after SFTSV infection and drug treatment, were fixed with 4% paraformaldehyde and permeabilized with 0.5% Triton X-100. Next, the cells were blocked with 2% nonfat milk and stained with rabbit anti-NP polyclonal antibodies prepared by our laboratory and anti-rabbit 488-conjugated secondary antibodies (Abcam, UK). Cell nuclei were stained with DAPI (Sigma-Aldrich, Shanghai). Images were captured by a Cytation Image Reader I (BioTek, USA).

### Time-of-addition experiment

The treatments of the virus and cells with compounds were divided into four patterns. Pretreatment of the virus: the compounds were preincubated with the viruses for 1 hour, and then the compound-virus mixture was added to the cells to infect for 1 hour. The mixture was completely removed, and the cell supernatants were collected for detection 34 hours later. Pretreatment of the cells: the compounds were preincubated with the cells for 1 hour, then the viruses were added to infect for 1 hour, and the subsequent procedures were the same as those for the pretreatment of the virus. Treatment post infection: the cells were first infected by the viruses for 1 hour, then the supernatants were completely removed, the compounds were added, and the cultures were incubated for 34 hours until supernatant collection. Full-time treatment: the cells were pretreated with the compounds for 1 hour, then the viruses were added for infection for 1 hour, and the medium was replaced with fresh medium containing the corresponding compounds. The supernatants were collected 34 hours later. The compounds included tanshinone I (15 µM), emodin (15 µM), licoisoflavone A (15 µM), glycycoumarin (20 µM), licoflavone A (15 µM), and licochalcone D (20 µM). Favipiravir (T-705, 300 µM) was used as a control.

### Molecular docking

The 3D structurespatial data format (SDF) files of tanshinone I, tanshinone IIA, and BXA was downloaded from PubChem. Open Babel was used to generate PDBQT files of these ligands from SDF files. The L EndoN domain structures of SFTSV (6NTV), H1N1 (5DES), and LCMV (3JSB) were downloaded from the RCSB PDB. The AutoDock tool was used to convert these receptor structure files into the PDBQT format. Then, AutoDock Vina (v1.2.3) (42) was employed for docking the ligands to the L EndoN domain. The grid box was set as a cube with a side length parameter of 30 and centered on the EndoN active sites. To find a lower-affinity binding position, the exhaustiveness parameter was set to 32. The binding sites identified by the docking analysis were analyzed by the PLIP web tool (43) and visualized using PyMOL.

### Structure alignment of L endonuclease domains

The structural similarity of the L EndoN domains of SFTSV, H1N1, and LCMV was compared using an online tool (pairwise structure alignment) of the RCSB PDB (https://www.rcsb.org/alignment). SFTSV (6NTV) was used as a reference; H1N1 (5DES) and LCMV (3JSB) were also submitted. The alignment parameters were set as flexible jFATCAT. Nuclease superfamily analysis of the L EndoN domains of SFTSV (YP_006504091.1), HRTV (YP_009047242.1), GTV (YP_009666941.1), LCMV (AFH08746.1), and H1N1 (AEA02268.1) was performed by alignment via a website (espript.ibcp.fr) with the ADV pattern.

### Endonuclease activity detected by gel electrophoresis

SFTSV EndoN protein was recombinantly expressed in *Escherichia coli* (BL21) and purified with Ni resin. The EndoN assay was carried out as previously described (44). Briefly, samples containing 20 mM Tris-HCl (pH 8.0), 150 mM NaCl, 1 mM TCEP, 1 mM MnCl_2_, and 5 µM EndoN protein were preincubated with tanshinone I (0.5 mM) or tanshinone IIA (2 mM) at 37°C for 30 minutes. BXA (10 mM) and DMSO (10%) were used as the positive and negative controls, respectively. The reaction was initiated by adding 0.5 µM ssRNA substrate (5′-FAM-AUUUUGUUUUUAAUAUUUC-3′) (31) synthesized by Tsingke (China). The reaction mixtures (40 µL) were incubated at 37°C, and the reaction was stopped by adding loading buffer (formamide containing 10 mM EDTA in 40 µL) and then heating at 95°C for 8 minutes. The products were then analyzed using 20% polyacrylamide/8 M urea gels. The images were visualized by Image Lab (Bio-Rad, USA) and analyzed by ImageJ software.

### Endonuclease activity detected by FRET

The enzymatic activity of SFTSV, IAV, and LCMV EndoNs was measured by a FRET-based endonuclease assay (45), with minor modifications. Briefly, SFTSV EndoN protein (1 µM) was mixed with 100 nM ssRNA substrate (5′FAM-AGGAAGAUUAAUAAUUUUCCU-BHQ1-3′) (Sangon Biotech, China) in a reaction buffer containing 50 mM HEPES pH 7.8, 150 mM KCl, and 1 mM MnCl_2_. The reactions were monitored by detecting fluorescence intensity (*λ*_ex_/*λ*_em_=485 nm/535 nm) on a microplate reader (SYNERGY H1, BioTek, USA). In the EndoN inhibition assay, the inhibitors were added at the indicated concentrations, and the reactions were monitored every 2 minutes until the signals were saturated. In contrast, the reaction system of IAV EndoN was 0.8 µM protein with 300 nM ssRNA substrate and that of LCMV EndoN was 0.4 µM protein with 400 nM ssRNA substrate. The inhibition of enzymatic activity by the compounds was calculated from the relative fluorescence units at saturation.

### Biolayer interferometry

Before starting the experiment, SFTSV EndoN proteins were first labeled with biotin at a molar ratio of 1:3 at room temperature for 0.5 hours, then filtered with a 3-kDa ultrafiltration tube to completely remove residual biotin. In a formal experiment, the biotin-labeled SFTSV EndoN protein was first diluted with reaction buffer containing 50 mM HEPES pH 7.8, 150 mM KCl, and 1 mM MnCl_2_ to 100 ng/mL. Tanshinone I and IIA were also diluted with the reaction buffer to 200, 100, 50, and 25 µM, or 800, 200, 100, and 50 µM, respectively. The detection was performed on an Octet RED96e (Sartorius, German) with a super streptavidin biosensor. The association time was set as 120 seconds, and the dissociation time was set as 180 seconds. Data were analyzed and calculated on ForteBio Data Analysis (v11.1), and the fitted curves were drawn by GraphPad Prism 8.0.

### *In vivo* challenge study

C57BL/6J mice (female, 8-weeks old) and BALB/c mice (male, 4 or 8 weeks old) purchased from GemPharmatech (Nanjing, China) were maintained in a specific pathogen-free animal facility and challenged in an animal biosafety level 2 (ABSL-2) laboratory.

For SFTSV challenge experiments, C57BL/6J mice were randomly divided into five groups (*n* = 4) and subjected to 20 mg/kg tanshinone I administration via three routes, i.g. administration, i.p. injection, and tail vein i.v. injection; the solvent and T-705 (300 mg/kg, i.g., once a day) were made as controls. The compounds were delivered 2 hours after challenge with SFTSV (5 × 10^5^ FFUs) or daily, and the body weight was monitored. Alternatively, tanshinone IIA was delivered via i.v. injection at 50 mg/kg 2 hours after challenge with SFTSV (5 × 10^5^ FFUs) via i.p. injection. T-705 (150 mg/kg, i.g., twice a day) delivered via i.g. administration was used as the control. Three days later, the mice were sacrificed for viral detection. Blood, spleens, or kidneys were collected from each mouse for viral copy detection or proinflammatory cytokine analysis.

For IAV challenge experiments, 4-week-old BALB/c mice were randomly divided into six groups (two in the mock group and four in other groups). Tanshinone I was delivered via i.v. administration at 5, 10, or 20 mg/kg after challenge with IAV (H1N1, 1,000 PFUs) via the i.n. route. For LCMV challenge experiments, 8-week-old BALB/c mice were treated with tanshinone I via i.v. administration at 5 mg/kg after challenge with LCMV (Armstrong, 2 × 10^5^ FFUs) via i.p. administration. The compounds were delivered daily, and the body weight was monitored daily. T-705 (300 mg/kg, i.g., once a day) delivered via i.g. administration was used as the control. Three days later, the mice were sacrificed for viral detection. Lungs or livers were collected from each mouse for viral copy detection, proinflammatory cytokine analysis, or pathological evaluation.

### Quantification of viral copies and proinflammatory cytokines from mouse tissues

The spleen, kidney, lung, or liver tissues removed by dissection were weighed and then ground in 1 mL of DMEM by a homogenizer at 60 Hz for 45 seconds three times at 15-second intervals. Total RNA was extracted from 200 µL of homogenate or 100 µL of blood according to the protocol of the RNeasy Mini Kit (Qiagen, Germany). The viral copies of SFTSV, IAV, or LCMV were quantified according to the procedure mentioned above in the cell experiments by qRT-PCR with the standard curve method. The relative levels of proinflammatory factors were measured by qRT-PCR with the _ΔΔ_Ct method. Mouse ACTB was used as the housekeeping gene, and the primers for proinflammatory factors are listed in Table S1.

### Pathological analysis

Lung tissues fixed in 4% paraformaldehyde for more than 24 hours were dehydrated into wax with a gradient of alcohol. The wax-impregnated tissues were embedded, and the wax blocks were placed on a paraffin microtome to form 4 µm sections. Paraffin sections were dewaxed in water and stained with hematoxylin for nuclei and eosin for cytoplasm (H&E). The sections were dehydrated in alcohol and xylene and finally sealed with neutral gum. Images (10× and 200×) were captured by microscopy (Nikon Eclipse CI) and processed with the Nikon DS-U3 system. Pathological scores were evaluated from each section.

### Pharmacokinetic analysis of tanshinone I in rats

Male Sprague-Dawley rats (180–220 g) were randomly divided into three groups (i.g., i.p., and i.v.) with three in each group. All rats were fasted overnight with free access to water prior to tanshinone I administration. Tanshinone I, which was suspended in 0.5% sodium carboxymethylcellulose, was orally administered by gavage at a dose of 20 mg/kg. The same dose of tanshinone I dissolved in 5% DMSO with solutizers as 5% Solutol HS-15 and 5% PEG-400 was administered via i.p. or i.v. Plasma samples were collected from the shoulder and jugular vein from 0 to 24 hours. Twenty-five microliters of plasma were mixed with equal volume of diclofenac sodium and then vortexed for 3 minutes, then 200 µL acetonitrile was added and vortexed for 5 minutes. The mixture was centrifuged at 1,700 g for 15 minutes at 4°C; 150 µL of supernatant was separated and mixed with equal volume of ultrapure water for analysis. The plasma concentration of tanshinone I at each time point was detected by ultra high pressure liquid chromatography with an LC-MS/MS system and calculated with standard curve method.

### Data analysis

Data are represented as the means ± SEMs from at least three independent replicates, except those specifically noted. The graphs, histograms, and heatmaps were produced with GraphPad Prism 8.0, and the statistical analysis was also performed with GraphPad Prism 8.0. The statistical significance of differences between two groups was determined using Student’s *t*-test with unpaired Mann-Whitney test. *P* values were defined as *ns. P* > 0.05, *P < 0.05, **P < 0.01, and ***P < 0.001.

## Data Availability

All data supporting the findings of this study are available within the article and supplemental materials, or from the corresponding author upon reasonable request.

## SUPPLEMENTAL MATERIAL

Supplemental materials jvi.00796-23-s0001.docx Supplemental figures and table.

ASM does not own the copyrights to Supplemental Material that may be linked to, or accessed through, an article. The authors have granted ASM a non-exclusive, world-wide license to publish the Supplemental Material files. Please contact the corresponding author directly for reuse.
