# Supplementary material for: Identification of tanshinone I as cap-dependent endonuclease inhibitor with broad-spectrum antiviral effect
Source: J Virol. 2023 Sep 21;97(10):e00796-23. doi: 10.1128/jvi.00796-23 (PMC10617418; doi:10.1128/jvi.00796-23)
Supplement: Supplemental materials — Supplemental figures and table. [file jvi.00796-23-s0001.docx]

Identification of tanshinone I as cap-dependent endonuclease inhibitor with broad-spectrum antiviral effect

Xiaoxue He^a^, Fan Yang^b^, Yan Wu^a^, Jia Lu^a,c^, Xiao Gao^a,c^, Xuerui Zhu^a^, Jie Yang^d^, Shuwen Liu^e^, Gengfu Xiao^a,c^, Xiaoyan Pan^a,c#^

Running title: Identification of tanshinone I as broad-spectrum CENi

^a^State Key Laboratory of Virology, Wuhan Institute of Virology, Center for Biosafety Mega-Science, Chinese Academy of Sciences, Wuhan, China;

^b^The Second Clinical Medical College, Jinan University (Shenzhen People's Hospital), Shenzhen, China;

^c^University of the Chinese Academy of Sciences, Beijing, China;

^d^NMPA Key Laboratory for Research and Evaluation of Drug Metabolism, Guangdong Provincial Key Laboratory of New Drug Screening, School of Pharmaceutical Sciences, Southern Medical University, Guangzhou, China;

^e^Guangdong Provincial Key Laboratory of New Drug Screening, Guangzhou Key Laboratory of Drug Research for Emerging Virus Prevention and Treatment, School of Pharmaceutical Sciences, Southern Medical University, Guangzhou, China.

^#^To whom correspondence should be addressed: Xiaoyan Pan, [panxy@wh.iov.cn](mailto:xiaogf@wh.iov.cn).

**Supplementary materials**


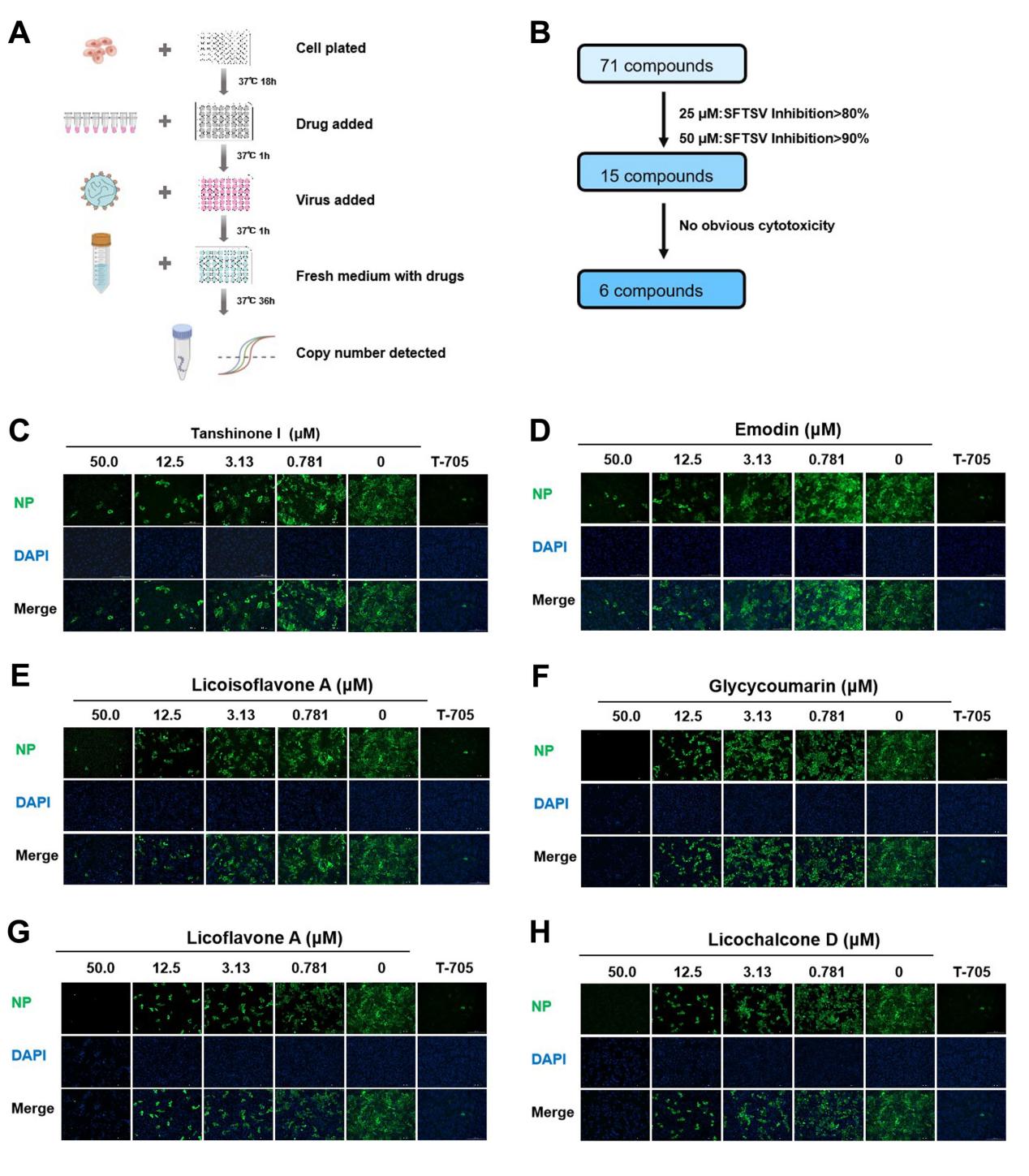


**Fig s1** **Screening of hit compounds from a mini natural product library against SFTSV.** **A** The scheme of screening process. **B** Flowchart of screening standard and the results. **C, D, E, F, G** and **H** IFA of the SFTSV infection on Vero cells after treatments with tanshinone I, emodin, licoisoflavone A, glycycoumarin, licoflavone A, and licochalcone D 36 hours post infection. T-705 (300 μM) was made as a control. SFTSV was stained with the rabbit polyclonal antibodies against NP and a second antibody conjugated with FITC, the nucleus was stained with DAPI.


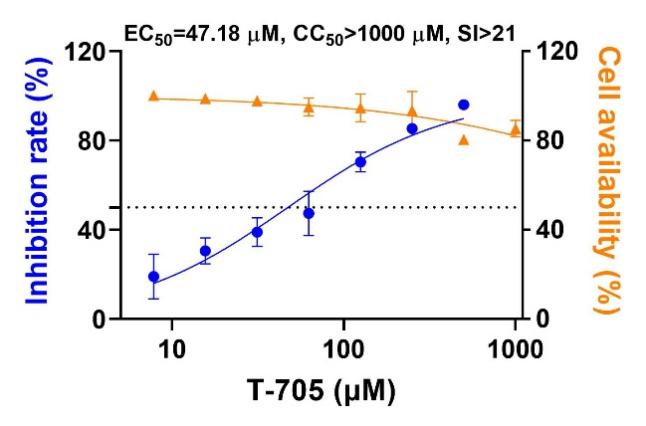


**Fig s2 The anti-SFTSV effect and cytotoxicity of T-705 on Vero cells.**

**
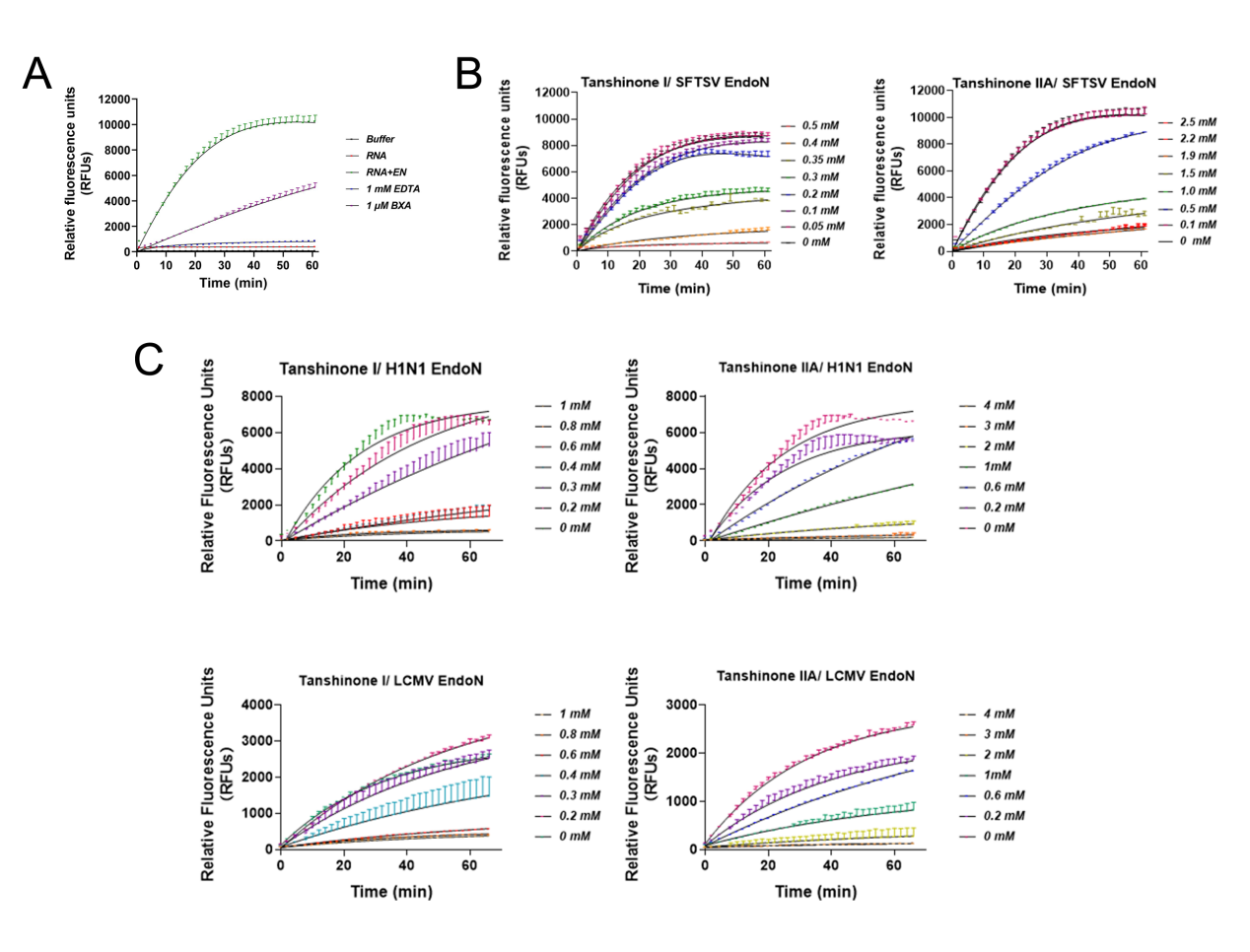
**

**Fig s3 Enzymatic activity detection by FRET.** **A** A test on the SFTSV EndoN enzymatic activity detection system by FRET. The reaction buffer was made as blank control, the RNA substrate was made as the background, RNA+EN was made as the negative control, EDTA and BXA were made as the system reference and positive control, respectively. **B** and **C** Dose-dependent inhibition of EndoNs from SFTSV, IAV and LCMV by tanshinone I and IIA was detected by FRET assay.


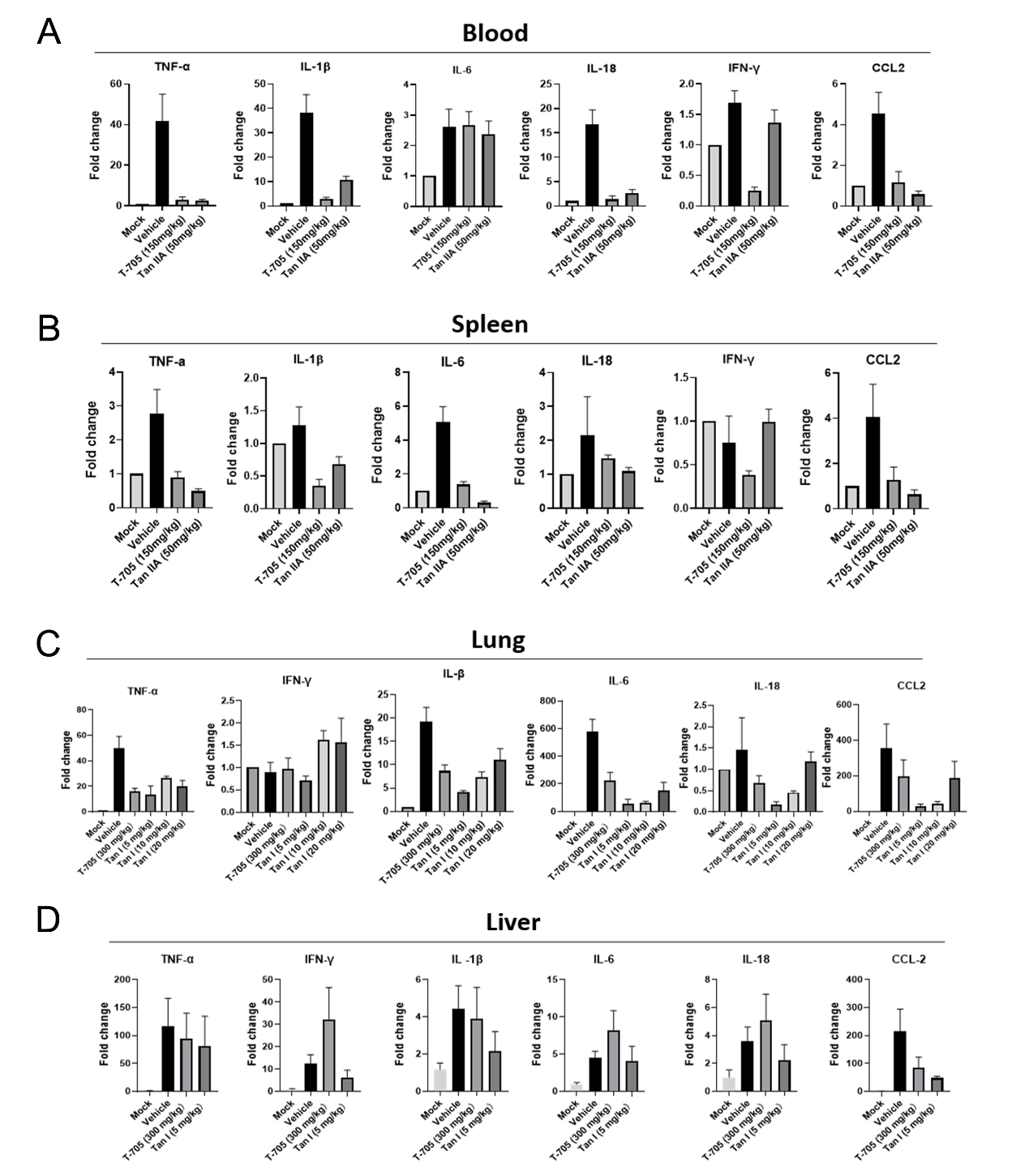
**Fig s4 Expression levels of proinflammatory cytokines in mouse tissues.** **A** and **B** The relative expression of proinflammatory cytokines in spleen and blood of C57BL/6J was linked to **Fig. 8E** and **8F**. **C** and **D** The relative expression of proinflammatory cytokines in lung and liver of BALB/c was linked to **Fig. 9B** and **9C.**


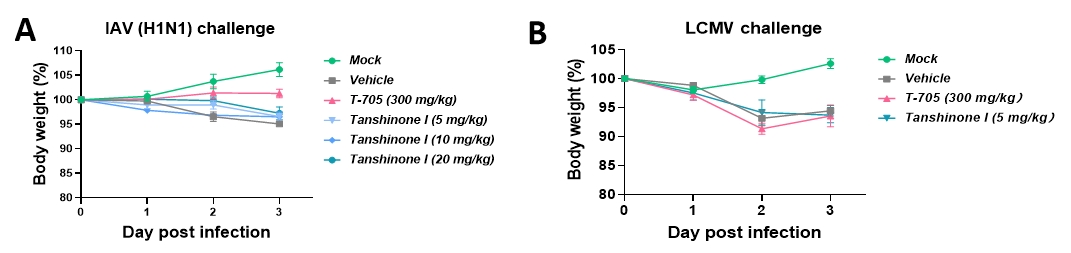


**Fig s5 The body weight** **curves after challenge by IAV and LCMV.**

**Table s1 The primers for qRT-PCR**

| **Primer** | **Sequence** |
| --- | --- |
| **SFTSV-NP-F** | TGCCTTCACCAAGACTATCAATGT |
| **SFTSV-NP-R** | GGGTCCCTGAAGGAGTTGTAAA |
| **SFTSV-RDRP-F** | ATYCACACMAGAYTRGATGGAAC |
| **SFTSV-RDRP-R** | TWTGCATCCATWGHTCTWGCCT |
| **LCMV-L-F** | GTAGTGGTCATGGCATCTTACC |
| **LCMV-L-R** | GAGAGGAGAAAGGCACCAATAG |
| **IAV-HA-F** | GGGTCCCTGAAGGAGTTGTAAA |
| **IAV-HA-R** | GAAGCAGTGGGTCGCATTCT |
| **β-ACTB-MICE-F** | TGTCCCTGTATGCCTCTGGT |
| **β-ACTB-MICE-R** | GATGTCACGCACGATTTCC |
| **TNF-α-MICE-F** | GCCTCTTCTCATTCCTGCTT |
| **TNF-α-MICE-R** | CACTTGGTGGTTTGCTACGA |
| **IFN-γ-MICE-F** | TGAAAGACAATCAGGCCATC |
| **IFN-γ-MICE-R** | TTGCTGTTGCTGAAGAAGGT |
| **IL-1β-MICE-F** | GAAATGCCACCTTTTGACAGTG |
| **IL-1β-MICE-R** | TGGATGCTCTCATCAGGACAG |
| **IL-6-MICE-F** | TTCCATCCAGTTGCCTTCTT |
| **IL-6-MICE-R** | ATTTCCACGATTTCCCAGAG |
| **IL-18-MICE-F** | GACAGCCTGTGTTCGAGGATATG |
| **IL-18-MICE-R** | TGTTCTTACAGGAGAGGGTAGAC |
| **CCL2-qF1** | CACCAGCCAACTCTCACTGAA |
| **CCL2-qR1** | GTGGGGCGTTAACTGCATCT |
